# The social accountability of doctors: a relationship based framework for understanding emergent community concepts of caring

**DOI:** 10.1186/s12913-017-2239-7

**Published:** 2017-04-12

**Authors:** Lionel P. Green-Thompson, Patricia McInerney, Bob Woollard

**Affiliations:** 1 Faculty of Health Sciences, University of the Witwatersrand, PV Tobias Health Sciences Building, 5 York Road, Parktown, 2193 Johannesburg, South Africa; 2 Department of Family Practice, University of British Columbia, Vancouver, Canada

## Abstract

**Background:**

Social accountability is defined as the responsibility of institutions to respond to the health priorities of a community. There is an international movement towards the education of health professionals who are accountable to communities. There is little evidence of how communities experience or articulate this accountability.

**Methods:**

In this grounded theory study eight community based focus group discussions were conducted in rural and urban South Africa to explore community members’ perceptions of the social accountability of doctors. The discussions were conducted across one urban and two rural provinces. Group discussions were recorded and transcribed verbatim.

**Results:**

Initial coding was done and three main themes emerged following data analysis: the consultation as a place of love and respect (participants have an expectation of care yet are often engaged with disregard); relationships of people and systems (participants reflect on their health priorities and the links with the social determinants of health) and Ubuntu as engagement of the community (reflected in their expectation of Ubuntu based relationships as well as part of the education system). These themes were related through a framework which integrates three levels of relationship:

- a central community of reciprocal relationships with the doctor-patient relationship as core;
- a level in which the systems of health and education interact and together with social determinants of health mediate the insertion of communities into a broader discourse.
- An ubuntu framing in which the tensions between vulnerability and power interact and reflect rights and responsibility. The space between these concepts is important for social accountability.

**Conclusion:**

Social accountability has been a concept better articulated by academics and centralized agencies. Communities bring a richer dimension to social accountability through their understanding of being human and caring. This study also creates the connection between ubuntu and social accountability and their mutual transformative capacity as agents for social justice

## Background

The World Health Organization (WHO) defined social accountability of educational institutions as early as 1995 [1]. The definition requires that these institutions define and respond to the community’s health priorities. These priorities should be jointly determined by various stakeholders including the community. This definition is complemented by the World Bank’s view that social accountability, through empowerment of communities, ensures they achieve sustainable development [2]. This development is enhanced when citizens have a direct engagement in defining health priorities and experience accountability from their service providers in the health care system, including doctors.

Woollard describes a partnership pentagram in which communities, policy makers, health professions and administrators as well as academic institutions relate to each other equally in an attempt to build a responsive health care system [3]. The four key areas in such a health system would be equity, cost effectiveness, quality and relevance [1].

While social accountability has taken an aspirational perspective within educational institutions, there is a critique that calls for the professions’ acknowledgement of the power they hold in health care [4]. This call highlights the need for greater critical reflexivity in the discourse around social accountability [4].

There have been many responses to the call for social accountability. The placement of medical students in community sites for their education has built close relationships with communities [5–9]. The symbiotic relationships which are developed between various stakeholders benefit the community and enhance students’ learning [7, 8]. However, in most cases, the relationships are not well defined and the voice of communities in understanding and defining those relationships has not been articulated in the literature.

Many communities host medical students for part of their curriculum in order to achieve their core competencies. The medical curriculum at the University of the Witwatersrand in Johannesburg, South Africa has committed to four competencies - provision of patient care in plural health and social contexts, developing and delivering appropriate care extending beyond the acute presentation of illness, delivery of effective care enhanced by cultural safety and social awareness and the competency to deliver primary care in defined geographical communities (University of the Witwatersrand, 2003). The university achieves these competencies by rotating students through clinical facilities and communities in the central urban Gauteng province and the rural provinces of Mpumalanga and North West.

This study aimed to give expression to the voices of these communities through exploration of their perceptions and understanding of the social accountability of doctors.

## Methods

The study was approved by the University Human Research Ethics Committee (Clearance M120695). For this study, a community was defined as a residential settlement which was served by any of the clinical facilities through which students from the university rotated for their studies. These communities were located in the North West, Mpumalanga and Gauteng provinces of South Africa. These were selected because the time that students spent in these areas ensured the communities were aware of the university’s presence in that region. Members of the community were identified through a research assistant. The research assistant, who was present during the discussions, was employed in each of the communities to assist with identifying participants who were not actively in need of acute care at the time of the study and who would facilitate reflexivity in the discussions [10, 11].

The group discussions were held in various locations such as a participant’s home, a school, a church building and a research centre which was not directly associated with the health facility at which participants received their regular health care. These were conducted between December 2012 and August 2013.

A grounded theory approach was employed using eight community based focus groups. Five focus groups were held in North West and Mpumalanga (Rural 1–5) followed by the three focus group discussions (Urban 1–3) held in Gauteng. These are provinces in South Africa. There were a total 81 community members who took part in the focus group discussions of whom the majority (63) were women.

Participants received a stipend for travel costs incurred by their participation in these groups and refreshments were supplied before and after the focus group discussions.

The focus group discussions took 45–60 min each and were guided by a series of questions with exploration of the meaning of each response (See Table 1).

**Table 1:** Questions which guided community focus group discussions

1. Tell me about your experiences of and with doctors?	
2. As a member of the community, what do you expect of your doctors?	
3. What, if anything, do you know about the social accountability of doctors?	
4. Do you think that doctors should be accountable to communities? If so, how do you think this should happen?	
5. What do you think are the major health issues in your community?	
6. Do you think that doctors need to get involved in issues in the community?	

The focus group discussions were recorded and transcribed verbatim.

The first author (LGT) is a male specialist clinician with an appreciation of the role which communities can play in medical education. This researcher moderated the discussions. He is also conversant with some of the vernacular languages in these areas and used the research assistant for basic translation occasionally during the discussions. The second author (PMcI) is a female nurse-midwife with a broad experience of health sciences education. The third author (BW) is an international authority in social accountability. All authors had experience in qualitative research while this study formed part of the doctoral studies of LGT. The data were coded using MAXQDA 11 software by LGT. PMcI co- coded the data. Constant comparison of the data after each discussion facilitated the recognition of themes from the coded transcripts [12–14]. Thematic analysis was performed following each discussion and saturation of data was achieved by the end of the eight discussions [12, 15]. Trustworthiness of the data was ensured through the checking of the transcriptions and the contemporaneous recording of field notes following each discussion. The groups could not be reconvened and so member checking was done through the continuous checking within the group discussion and constant comparison in subsequent focus group discussions.

The participants in the first two group discussions (Rural 1 and 2) were unable to understand the meaning of social accountability of doctors (Question 3). As a result, the researcher (LGT) did not ask this question directly in subsequent discussions but rather explored the first two responses in greater detail in order to understand the community’s expectation of the doctor. In the earlier discussions, the participants raised the expectation that the university should play a greater role in the life of the community. This was explicitly explored in all the subsequent discussions. The views expressed led to discussion and consensus within the group. Focus groups were conducted so that all participants contributed to the discussions and all contributed freely to the discussions. Extracts are labelled for the group from which each emerged.

## Results

Community participants were unable to articulate a definition of social accountability or suggest how they could hold doctors accountable. They value the role of the doctors in their wellbeing but feel powerless to demand any form of accountability. Community experiences were measured most profoundly by the achievement of a meaningful relationship with the caring doctor.

Three major themes emerged from the coded data in which the consultation was characterised as a *place of love and respect* at the heart of *the relationships amongst people and systems.* Both of these were surrounded by the notions of *Ubuntu as engagement with community.*

### The clinical consultation as a place of love and respect

Participants acknowledged that their encounters with doctors happened when they were most vulnerable. The consultation, as a result, was experienced as both a place of healing and a place of danger. The participants’ descriptions of these encounters evoked the tension between their tenderness and danger of their disease:

*You see they are supposed to touch with love*, *because inside (the body) it’s very dangerous (Rural 2).*

The healing which participants expect from their consultations was made up of two dimensions: sound relationships which were therapeutic and, only secondarily, the dispensing of appropriate treatments.

*When I come to the doctor, I have an illness problem. I want to get here, sit down and the doctor listens to me and then after listening if the doctor does not understand what I’m saying then he can have all the apparatus they use to check me as it is necessary for him, with care and love (Rural 2).*

These sound relationships reflect their desire for respect and dignity. The notion of love in a consultation was expressed in the following:

*Love is when you get a treatment and the doctor explains it in the way you understand it (Rural 5).*

The participants anticipate mutual responsibility within the consultation where the doctor may collaborate with them, sharing responsibility for the solution of the presenting problem. The participants described the outcomes from the consultation as being a product of things the doctor does, things the patient does and the many things which occupy the relationship space between a patient and their doctor. The doctor’s role and the patient’s role are mediated through an emotive space – characterised by pain and fear, vulnerability, love and care - which exists between them in a consultation. This asymmetrical relationship is imbued with great power differentials.

The power accorded to doctors appears to be a function of patients’ being *afraid to confront the doctor (Urban 2)* because of the knowledge which the doctor has accumulated through university study. The consequence of this impotence is that *you (the patient) put him (the doctor) on a pedestal (Urban 2).* The doctor is placed in a space which is other than that inhabited by the patient.

### Relationships of people and systems

#### The people

**Fig. 1:**
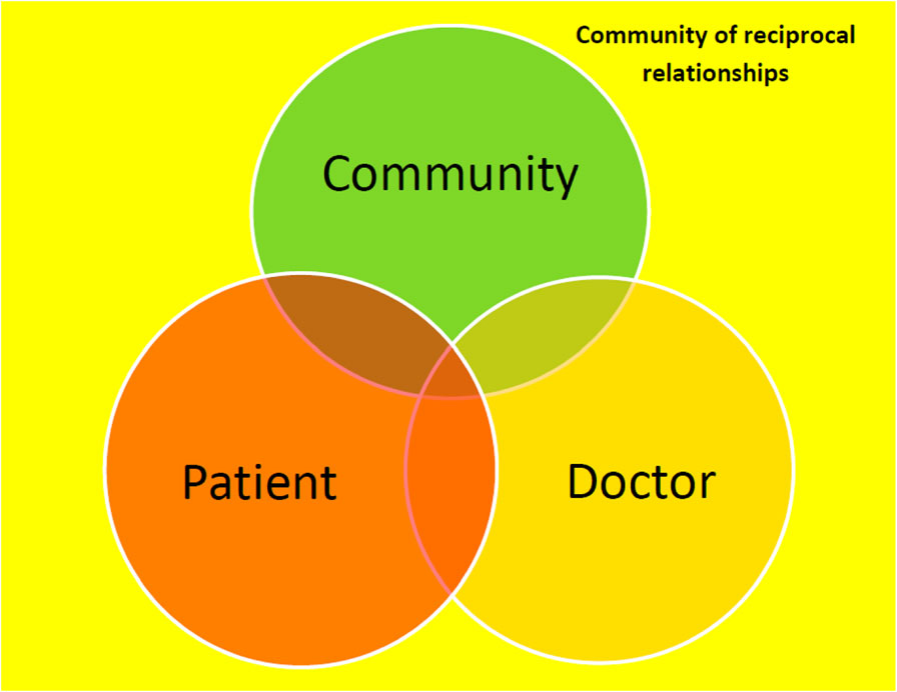
Community of reciprocal relationships

There was a strong sense that doctors are not part of the communities in which they work. Participants describe various relationships: between the doctor and the patient, between the patient and the community and between the doctor and the community. This last relationship is integral to the empowerment for the community. All of these relationships are dynamic and form a reciprocal community of relationships amongst all these actors. There is an opportunity for patients, doctors and well members of communities to interact to improve the health of the community as suggested by Fig. 1.

Participants had low expectations of the doctors’ immersion in their communities but there was an expectation that doctors should help communities do things for themselves:

*Doctors, the one thing they should be doing, is to alert the community for the community to take responsibility to take care of themselves (Rural 5)*

Community participants listed a series of biomedical conditions as their health priorities: diabetes mellitus, hypertension, cancer, strokes, HIV and sexually transmitted infections, tuberculosis and industrial exposures causing illness in children. In addition, teenage pregnancies and crime and violence associated with substance abuse form a part of communities’ disease profiles.

In addition, they described strong links between these diseases and the poor social conditions under which they live:

*You know poverty causes a lot of things; stress, unhealthy decisions. So most definitely I think poverty plays a big role (Urban 3).*

Despite this there was a strong sense that communities can be developed to take on the responsibility for addressing the health issues that arise. There was a call for the strengthening of the role of the doctor in empowering communities to work on the social determinants of their health.

Multiple dynamic reciprocal relationships impact on the community’s experience of the health care system and on their state of health (Fig. 1). The doctor – patient relationship remains central to the therapeutic process (*the conversation I have with the doctors, (it) helps me to be healed without any treatment - Rural 2).* This relationship forms the centre of a broader view of the role of the doctor as an agent of community empowerment.

#### The systems

Participants reflected on doctors whom they had encountered in both private and public health care settings as being part of complex systems with many parts and many actors (professionals and support workers)..

Many of the groups stated that financial incentives dominated private practice ensuring greater responsiveness of doctors in that sector. Communities expressed willingness to be part of change processes but expected similar responsiveness to their needs within the public system as well. They believed that solutions could only emerge from involvement of all levels of the health system, particularly, the management.

Continuity of care was seen as important for accountability. Participants who saw the same doctor on more than one occasion in the public sector regarded themselves as *lucky (Urban 2).* This was supported by the concerned comment in one of the discussions:

*Tomorrow when you come maybe there is another doctor and you don’t know the doctor you see (Urban 3).*

The reciprocal community of relationships (Fig. 1) is in constant engagement with a more universal context in which power, rights and responsibility interact with each other. Within this context these relationships encounter:

- the health care system (*they (doctors) don’t use Batho Pele(SeSotho word meaning people first) principles - privacy, confidentiality of the patient (Rural 2)*
- the social determinants of health – poverty (*poverty causes a lot of things; stress, unhealthy decisions … we don’t eat healthily – Urban 3)* and agency (*we as the community, it’s us who cause all these things – Urban 3*).
- the education system. (*I think in as much as they study medicine they must study people and the community -Rural 1*).

Batho Pele embodies a series of values (service excellence, consultation with stakeholders, courtesy, access, information, openness and transparency, redress and cost efficiency) which the public service in South Africa made a commitment to uphold. In the health sector this translates into the responsibility for the achievement of health for the broader community being shared by all stakeholders – communities, doctors and systems of health and education. The systems which mediate the achievement of health reflect the community’s concepts of social accountability. Participants reflected on the importance of relationships in all these interactions..

### Ubuntu as engagement of the community

Ubuntu, as a manifestation of African humanism, is characterised by the relatedness of humanity as suggested in the Zulu aphorism: *umuntu ngumuntu ngabantu* (a person is a person through other people) [16]. Ubuntu’s many facets of respect, dignity, solidarity, compassion and survival all contribute to the connections amongst people as well as their connections to the systems in which they relate [17].

Two dimensions of ubuntu were expressed by community participants. These dimensions appear to be a combination of the doctor – community relationship as well as a humanistic character of the practitioner *(… to have humility. To know that you are dealing with a human being who is not feeling well and you are there (Urban 2).*

Participants suggest that the university is responsible for their graduates’ behaviour:

*… when you (refers to university) are teaching these doctors you need to teach them ubuntu as well, so that they know what to do when they come to communities (Rural 2).*

In teaching this concept of ubuntu, the community expected that students would emerge from their training with knowledge beyond their biomedical information:

*I think in as much as they study medicine they must study people and the community (Rural 1).*

Knowing and understanding the community would enhance the graduates’ capacity for individual and communal health care. Participants felt that the university was currently largely unsuccessful in achieving this level of *“study (of) community”*. In an urban area (Urban 2) where the university appoints the medical staff jointly with the provincial authority, participants did not acknowledge that the local hospital represented a joint endeavour between the university and the provincial government. In one rural community group (Rural 3), the university was seen as valuing indigenous forms of healing. The involvement of traditional healers in the undergraduate medical programme facilitated the emergence of these practitioners: *now we have come out of our rondavels (traditional dwellings in South Africa) so that everybody can know us because of Wits(the university) (Rural 3).*

This emergence from a traditional space suggests an engagement between the university and its reference population which has already developed in a spirit of co-responsibility. The participants described the participation of doctors in the general life of the community as assisting those communities to take greater responsibility for their health.

Ubuntu has been described as both a positive characteristic within the individual healing medical encounter (*to have humility – Urban 2*) as well as an essential part of the education of future doctors (*you need to teach them ubuntu as well – Rural 2*). These conceptions of ubuntu are essentially relationship based ideas.

The framework of ubuntu is echoed in the partnership pentagram where an ever widening circle of impact is reflected from a local to a national level [3].

**Fig. 2:**
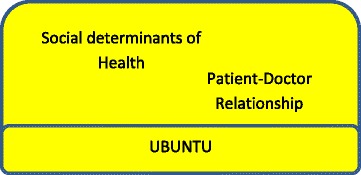
Ubuntu as the foundation of empowered community relationships

The humanistic ideal of ubuntu suggests that the patient – doctor relationship is a key element in understanding both the vulnerability of the attending patient as well as the power held by the practitioner. Interestingly, a sense of taking responsibility has emerged from the data suggesting an explicit role for community or patient empowerment in addressing the social determinants of health. This important finding offers an opportunity to rethink policies and practices of health systems that seem to assume, and even reinforce, a sense of “learned helplessness” of the population in program development. Properly engaged this empowerment amongst communities and in their relationships with doctors will shift them towards an attainment of their right to good health and their shared responsibility for maintaining the state of their health. This Ubuntu approach has been described by participants as the foundational concept supporting a positive interaction between the reciprocal relationships and the social determinants of a community’s health (see Fig. 2).

## Discussion

The doctor holds immense power in the clinical consultations with individual patients as well as within communities. Despite the asymmetrical nature of this relationship, ubuntu within which the patient – doctor relationship may reside is a foundational element for a socially accountable system. This study has articulated the tension which exists within the clinical consultation between the patient’s vulnerability and their desire for co-responsibility with the doctor. Malena et al. [2] have described the interaction between the service provider and the community as an important accountability relationship*.* Despite the best intentions of transforming environments to achieve this situation, the poor often encounter both health professionals and front line health managers whose behaviour may result in injustice for ordinary people [18, 19]. It is often the poor who are disadvantaged in the systems which seek their development [2].

Ubuntu supports the community of reciprocal relationships as the keystone to socially accountable medical practice. Ubuntu frames the local and global experience in which the social determinants of health and the systems of health care and education interact (Fig. 2).

The communities in this study show a good level of understanding of their health priorities. These are similar to national statements on the burden of disease [20]. They also highlighted the links between these priorities and their social conditions related to poverty and social breakdown. The understanding and attitudes of patients and community members reflects an untapped resource for focused change in order to empower communities to take greater responsibility for their health.. While not necessarily being expressed in terms of “social accountability”, it is clear that patients and citizens voice an understanding of the required resilience to be active and effective “pentagram partners” in building a health care system based on people’s health priorities—the essence of social accountability. In addressing health there needs to be a greater focus on a range of social determinants such as employment, education and social protection in order to close the equity divide between wealthy and poor communities [21]. The study of communities (and perhaps those like them from around the world) is a good place to start.

The emergent concepts of relationships (amongst people and between systems) from this study are echoed in Worley’s description of the symbiotic relationships which occur in teaching environments which may enhance social accountability [7, 8]. Hirsh and Worley [22] in conversation with each other place great emphasis on the role of a different structure of education, based in part on symbiotic models, as promoting the transformative nature of the graduate which emerges.

This study adds to our understanding by adopting the concept of ubuntu as a richer expression of humans caring for one another. These intersecting and symbiotic relationships reflect a dimension of ubuntu or African humanism [17]. Mbigi [17] posits that ubuntu in a transformative management environment reflects five dimensions of human interaction, namely, survival, dignity, respect, compassion and solidarity. Khoza’s view of ubuntu as a dimension of African humanism echoes the need of the individual for dignity, self-respect and regard for others [23].

The community’s reflections in this study have highlighted this sense of Ubuntu. They have described it as an essential part of the consultation but, more importantly, of the university’s role in relationship with the community.

Ubuntu may facilitate the transformation of the traditional relationship between the patient (vulnerable) and the doctor (powerful). It may help to create a space where things often left unspoken in asymmetrical relationships may gain a voice.

## Conclusion

Social accountability has emerged in this study as a concept which is better understood by academics and centralized agencies. On the other hand, the communities have brought a richer understanding of what it means to be both human and caring. The reflection of these communities has located the definition firmly in the context of relationships. Ritz et al have argued that we will need to acknowledge more clearly that social accountability must be defined by communities as genuine partners in systems transformation [4]. To this end, social accountability and ubuntu may become the transforming agents for social justice as originally intended.
